# Case of Poisoning by Opium

**Published:** 1854-01

**Authors:** James M. Nye

**Affiliations:** Lynn, Mass.


[Fom the N. H. Journal of Medicine.

*Case of Poisoning by Opium.*—New Mode of Inducing Vomiting.

MR. EDITOR:—Mrs. S., æt. 30, previously in good health, while in a passion, induced by some domestic feud, procured and swallowed from ℨv to ℨviij. of tinct. opii. In a short time she became quite stupid. The poison had been swallowed forty-five minutes, when I arrived, and she had taken large draughts of tepid water, and mustard.

She was quite stupid; cerebral vessels distended; face flushed, and could walk only with the assistance of two persons. Wishes to be left alone, that she may go to sleep. While held erect, her head falls to one side, and she breathes with some difficulty.

Ordered an emetic of pul. ipecac and pul. sinapis, aa. ʒij. in tepid water, to be followed by several tumblers of water, the head to be showered with cold water. No vomiting occurred in thirty minutes. Repeated the emetic. Introduced a feather into the œsophagus. Waited fifteen minutes; no relief; symptoms more grave. Ordered two tumblers of diluted vinegar, to be immediately followed by ʒij. of carb. potassa, in water. A powerful effervescence took place, which instantly produced most copious vomiting.

This is a new remedy with me in such cases, and one attended with most desirable results. It may answer instead of the stomach pump in many cases. If you think the case of sufficient interest, it is at your service for the Journal.

JAMES M. NYE.

Lynn, Mass. Nov. 17, 1853.

---

(We recollect reading some years since, a case similar to the above; if our memory serves us right, in that case the patient, (from insensibility or obstinacy,) could not or would not swallow; the solution of alkali and the vinegar were injected into the Stomach through a tube; they were used in large quantities, and got up quite an impromptu "spouting fountain." In that case as in this, the result was satisfactory.—Ed.)

